# A hole inversion layer at the BiVO_4_/Bi_4_V_2_O_11_ interface produces a high tunable photovoltage for water splitting

**DOI:** 10.1038/srep31406

**Published:** 2016-08-09

**Authors:** Wayler S. dos Santos, Mariandry Rodriguez, André S. Afonso, João P. Mesquita, Lucas L. Nascimento, Antônio O. T. Patrocínio, Adilson C. Silva, Luiz C. A. Oliveira, José D. Fabris, Márcio C. Pereira

**Affiliations:** 1 Institute of Science, Engineering and Technology (ICET), Federal University of the Jequitinhonha and Mucuri Valleys (UFVJM), Campus Mucuri, 39803-371 Teófilo Otoni, Minas Gerais, Brazil; 2 Graduate Program in Biofuels, Federal University of the Jequitinhonha and Mucuri Valleys (UFVJM), Campus JK, 39100-000 Diamantina, Minas Gerais, Brazil; 3 Department of Chemistry, Federal University of the Jequitinhonha and Mucuri Valleys (UFVJM), Campus JK, 39100-000 Diamantina, Minas Gerais, Brazil; 4 Institute of Chemistry, Federal University of Uberlândia (UFU), 38400-902 Uberlândia, Minas Gerais, Brazil; 5 Institute of Exact and Biological Sciences, Federal University of Ouro Preto (UFOP), 35400-000 Ouro Preto, Minas Gerais, Brazil; 6 Department of Chemistry, Federal University of Minas Gerais (UFMG), 31270-901 Belo Horizonte, Minas Gerais, Brazil

## Abstract

The conversion of solar energy into hydrogen fuel by splitting water into photoelectrochemical cells (PEC) is an appealing strategy to store energy and minimize the extensive use of fossil fuels. The key requirement for efficient water splitting is producing a large band bending (photovoltage) at the semiconductor to improve the separation of the photogenerated charge carriers. Therefore, an attractive method consists in creating internal electrical fields inside the PEC to render more favorable band bending for water splitting. Coupling ferroelectric materials exhibiting spontaneous polarization with visible light photoactive semiconductors can be a likely approach to getting higher photovoltage outputs. The spontaneous electric polarization tends to promote the desirable separation of photogenerated electron- hole pairs and can produce photovoltages higher than that obtained from a conventional p-n heterojunction. Herein, we demonstrate that a hole inversion layer induced by a ferroelectric Bi_4_V_2_O_11_ perovskite at the n-type BiVO_4_ interface creates a virtual p-n junction with high photovoltage, which is suitable for water splitting. The photovoltage output can be boosted by changing the polarization by doping the ferroelectric material with tungsten in order to produce the relatively large photovoltage of 1.39 V, decreasing the surface recombination and enhancing the photocurrent as much as 180%.

The strategy based on storing solar energy in the form of chemical energy is the envisaged development of technology in an attempt to prevent or minimize harmful effects on the natural environmental, which has been recognizably accelerated by the large current use of fuels derived from fossil sources. Several recently reported studies have been focusing on the development of water-splitting photoelectrochemical (PEC) cells, which consists of devices assembled with semiconductor photoelectrodes that operate by using the sunlight radiation for producing H_2_ and O_2_ through the molecular splitting of water^1^,^2^. An input energy of 1.23 V is thermodynamically required for such a water splitting reaction and photoelectrodes with bandgap energies below 2.5 eV are needed for an efficient use of sunlight radiation^3^. To meet the best working conditions, the band edges potentials at the semiconductor surfaces must straddle the H_2_ and O_2_ redox potentials, the electrodes should be chemically stable regarding the photocorrosion, and the charge transfer from the surface of the semiconductor to the solution must be efficient enough to minimize the energy losses due to kinetics overpotentials^3^,^4^. To date, no known single material has intrinsic properties satisfying all these requirements. Coupling materials with synergic properties could be the most suitable way to design and build efficient photoelectrodes^5^. For example, it has been reportedly shown that coupling cocatalysts^6^,^7^, charge collectors^8^, and homo- or heterojunctions^9^,^10^,^11^,^12^ can improve the photocatalytic performance of nanomaterials.

In the more recent years, the n-type BiVO_4_ semiconductor has been widely studied as a photoanode in water-splitting PEC cells taking into account that it is composed of chemical elements that are relatively abundant on Earth, has a short bandgap energy (2.5 eV), its valence band is suitably positioned towards the water oxidation, and its conduction band is also suitably placed close to the H_2_ evolution potential^13^. However, the primary challenge for improving such photoactive BiVO_4_ consists in minimizing the fast electron-hole recombination due to the slow electron transport in BiVO_4_. Consequently, extremely low photovoltages (typically, V_ph_ < 0.6 V) have been reported for BiVO_4_^14^,^15^.

The more common strategy to increase the photoactivity of BiVO_4_ is based on the formation of p-n heterojunctions. Various p-n heterojunctions such as BiOI/BiVO_4_^16^,^17^, BiOCl/BiVO_4_^18^, Bi_2_O_3_/BiVO_4_^19^,^20^, NiO/BiVO_4_^21^, Si/BiVO_4_^22^, CuO/BiVO_4_^23^, Cu_2_O/BiVO_4_^24^, Ag_2_O/BiVO_4_^25^, and Co_3_O_4_/BiVO_4_^26^ have been proven to be an efficient method for keeping electron-hole pairs separated due to the built-in potential produced by the heterojunction. However, those mentioned p-type semiconductors are either unstable in water under oxidizing conditions or unable to absorb a wider visible light range than BiVO_4_, thus limiting its application on photoelectrochemical water splitting.

The water splitting process in PEC cells is driven by the band edge pinning effect, in which the band edge positions of a semiconductor are pinned about the water redox potential. Thus, the height of band bending and consequently, the V_ph_ can be estimated by the difference between the flatband potential (V_fb_) of the semiconductor and the chemical potential of the targeted reaction in water^27^. Regarding the BiVO_4_, the maximum theoretical photovoltage for the water oxidation is approximately 1.23 V based on its flatband potential of about 0 V_RHE_^7^,^13^. However, that theoretical V_ph_ value has not been reached up to now. We understand that the surface electronic states result in the pinning of the Fermi level of BiVO_4_, which decreases the experimental V_ph_ about the theoretical value^28^. To solve this, we can introduce internal electrical fields through ferroelectric polarization method by forming buried semiconductor/ferroelectric semiconductor heterojunctions.

For this purpose, in this study we made a heterojunction by coupling BiVO_4_ with Bi_4_V_2_O_11_ (Fig. 1), which is an n-type layered semiconductor that exhibits strong polar responses due to its polarized oxygen deficient perovskite slabs (*i.e.,* (VO_3.5 0.5_)^2−^; = anionic vacancy)^29^,^30^. When the ferroelectric Bi_4_V_2_O_11_ is coupled to the BiVO_4_, its spontaneous electrical polarization generates a sufficiently high electric field that produces an inversion layer at the BiVO_4_/Bi_4_V_2_O_11_ interface (Fig. 1a), thus resulting in an increased band bending, enhancing the charge generation and the separation efficiency. By this approach, BiVO_4_ is the light-absorbing material for generating the electron-hole pairs, whereas the ferroelectric Bi_4_V_2_O_11_ perovskite plays the role of absorbing light and creating a built-in electric field to separate the photogenerated charges, inducing the carrier transport. A key advantage of the BiVO_4_/Bi_4_V_2_O_11_ coupling junction is the appropriate energy band alignment (Fig. 1b), which facilitates the current flow^31^. We have shown that the photovoltage was readily tuned by doping the BiVO_4_/Bi_4_V_2_O_11_ heterojunction with tungsten ions. As a result, the photovoltage and photocurrent were enhanced as much as 83% and 180%, respectively.

## Results

### Photoelectrode synthesis and characterization

The XRD pattern of the sample Bi_4_V_2_O_11_ (Fig. S1a) confirmed the presence of Bi_4_V_2_O_11_ (*a* = 5.5479(5) Å, *b* = 5.5522(1) Å, and *c* = 15.4665(2) Å) without any impurity, while characteristics peaks of BiVO_4_ (*a* = 5.1808(2) Å, *b* = 5.0915(1) Å and *c* = 11.6801(1) Å) could be observed in Fig. S1b. The SnO_2_ peaks seen in the XRD patterns of these samples are due to fluorine-doped tin oxide (FTO). The BiVO_4_/Bi_4_V_2_O_11_ heterojunction prepared through the Pechini method was deposited onto FTO glass covered with SnO_2_ to act as a passivating layer by using the spray pyrolysis method as previously elsewhere reported^32^. The XRD pattern of the sample labeled W0 (sample of the undoped heterojunction) (Fig. S1c) revealed that the as-prepared film is constituted by the monoclinic scheelite BiVO_4_ phase (*a* = 5.1811(2) Å, *b* = 5.0912(1) Å and *c* = 11.6807(1) Å) and the orthorhombic Bi_4_V_2_O_11_ (*a* = 5.5488(5) Å, *b* = 5.5520(1) Å, and *c* = 15.4670(2) Å). To increase the photovoltage and facilitate the photocurrent, the tungsten was used as a dopant into the BiVO_4_/Bi_4_V_2_O_11_ film (sample labeled W1)^33^. The successful structural doping with W into the films was confirmed by the Rietveld analysis of the XRD data, which indicated the expanded unit cells for the W-doped BiVO_4_ (*a* = 5.1853(2) Å, *b* = 5.0931(2) Å and *c* = 11.6823(4) Å) and W-doped Bi_4_V_2_O_11_ (*a* = 5.5495(7) Å, *b* = 5.5528(1) and *c* = 15.4673(9) Å) (Fig. S1d). The quantitative Rietveld analysis showed that both heterojunctions are formed by 52 wt.% BiVO_4_ and 48 wt.% Bi_4_V_2_O_11_.

SEM images of the heterojunctions (Fig. S2) revealed the formation of BiVO_4_/Bi_4_V_2_O_11_ islands with a highly porous fiber-like morphology. The thickness of both heterojunction films determined by cross-sectional SEM was 9 μm (Fig. S3). The EDS mapping (Fig. S4) showed the uniformly distributed Bi and V elements throughout the films. In addition to Bi and V, it was also detected a homogeneous distribution of W all over the W1 heterojunction. The W content in the film was in average 1.2(3) wt.%, as determined by XRF analysis.

The DRS spectra (Fig. S5a) showed that the films absorb radiation over a wide range of wavelengths, from the UV to the visible regions of the optical spectrum. The optical band gap energies (Fig. S5b) estimated for the BiVO_4_ and Bi_4_V_2_O_11_ in the undoped heterojunction were 2.53 and 2.42 eV, respectively. This means that both components of the heterojunction can be excited by visible light to produce reactive species for the water splitting. The band gap energies of BiVO_4_ and Bi_4_V_2_O_11_ in the W-doped sample (W1 in the Fig. S5) are similar to those of the non-doped heterojunction. No visible impurity absorption exists as the W doping energy levels are shallow^34^.

### Photoelectrochemical performance

The current-potential curves presented in Fig. 2 clearly shows that the W doping of the BiVO_4_/Bi_4_V_2_O_11_ heterojunction improved the observed photocurrent of the photoelectrode under visible light (λ > 450 nm). Photocurrent values at 1.23 V_RHE_ were 0.05 and 0.14 mA cm^−2^ for the W0 and W1 heterojunctions, respectively. It has been reported that the replacement of V sites by W as dopant eliminates the hole traps and extend the electron lifetime, thus significantly increasing the photocurrent^33^. Indeed, the longer electron lifetime reflects the slower recombination process in W1 than W0 film (Fig. S6). Interestingly, the photocurrent values obtained with the BiVO_4_/Bi_4_V_2_O_11_ heterojunctions were significantly higher than those with pristine BiVO_4_ (0.012 mA cm^−2^) or Bi_4_V_2_O_11_ (9 × 10^−5^ mA cm^−2^) at 1.23 V *vs.* RHE (Fig. 2), indicating that the heterojunction formation enhances the charge separation and consequently the photocurrent. Corroborating to these data, the electrochemical impedance analysis (Fig. S7) showed that the hole-transfer resistance across the heterojunction-electrolyte interface is significantly smaller than those of pristine BiVO_4_ and Bi_4_V_2_O_11_, pointing out that the charge transfer processes on the BiVO_4_/Bi_4_V_2_O_11_ heterojunction are faster than those occurring on the surface of the individual components. Additionally, the charge transfer resistance across the W-doped heterojunction-electrolyte interface is approximately one-seventh relatively to the non-doped heterojunction, implying that the W doping can also significantly improve the charge transfer across the heterojunction-electrolyte interface. In other words, the charge transfer from the near surface region of the W-doped BiVO_4_/Bi_4_V_2_O_11_ heterojunction to the redox species on the surface is kinetically faster. Thus, as the charge transfer process becomes faster, it can efficiently minimize the electron-hole recombination at the BiVO_4_/Bi_4_V_2_O_11_ surface. As a result, the half-cell solar-to-hydrogen efficiency (HC-STH)^35^ in a three-electrode setup was substantially increased after the heterojunction formation and W-doping treatment. The HC-STH values at 0.85 V_RHE_ for the bare BiVO_4_, Bi_4_V_2_O_11_, W0, and W1 films were 0.06, 0.00, 0.27 and 0.60%, respectively (Fig. S8). The incident photon-to-current efficiency (IPCE) spectra in a two-electrode setup for front-side illuminated W0 and W1 heterojunction at the potential of 0 V_Pt_ (Fig. 3) showed a steady increase from 500 to 350 nm. The IPCE for the W1 film reached 2.6% at 450 nm with a maximum of 4.5% at 350 nm. On the other hand, the IPCE for the W0 film was significantly lower, reaching 1.0% at 450 nm and 1.4% at 340 nm at 0 V_Pt_.

### Open-circuit potential measurements

To better understand the effect of the heterojunction and W as a doping element on the enhancement of the photocurrent, we have tried to estimate the onset potential (V_on_) for each photoelectrode^36^. The V_on_ value for the pristine BiVO_4_ was 0.50 V_RHE_, whereas the heterojunction formation and W doping led to an increase of the V_on_ value. The V_on_ of 0.54 V_RHE_ was obtained for the W0 photoelectrode; after the W-doping, the V_on_ reached 0.66 V_RHE_ (Fig. S9). These trends are opposite to those obtained by electrochemical impedance (Fig. S7), which clearly showed that the W-doping into BiVO_4_/Bi_4_V_2_O_11_ significantly enhances the kinetics of charge transfer across the semiconductor-electrolyte interface by reducing the kinetic overpotentials. It suggests that the current *vs.* potential measurements are not suitable for quantitative analysis, as the change in V_on_ may be affected for both kinetics (reduction in kinetics overpotentials) and thermodynamic (increase in V_ph_) factors^37^. To evaluate which factor plays a more important role on improving the photocurrent, we then measured the open-circuit potential (OCP) in the dark and under visible light. It enabled us to probe the resting potentials at zero net exchange photocurrents, where the kinetic factors are negligible^37^. The difference between the measured potential in light and that in the dark led to the V_ph_. Hence, the pristine BiVO_4_ and Bi_4_V_2_O_11_ produced photovoltages of 0.11 and 0.13 V respectively, whereas the W0 and W1 heterojunctions gave the significantly higher photovoltages of 0.76 and 1.39 V, respectively (Fig. 4). The enlarged band bending at the BiVO_4_/Bi_4_V_2_O_11_ heterojunctions represent the enhanced electron-hole separation in the photoelectrodes. These data suggest that the heterojunction formation is essential to obtain high photovoltages. Moreover, the significant increase of 83% of the photovoltage value, compared to the non-doped heterojunction suggests that the W-doping treatment changes the BiVO_4_/Bi_4_V_2_O_11_ interface, thus increasing the degree of band bending. It is worth to note that the OCP in the dark usually reflects the upward band bending nature of the photoanode in dark equilibrium with the electrolyte. However, the significantly more positive OCP_dark_ values (1.8 V vs. RHE) were obtained with the heterojunctions, suggesting that the surface states of the BiVO_4_/Bi_4_V_2_O_11_ heterojunction were passivated to unpin the Fermi levels. As a result, an enlarged band bending was produced after the heterojunction formation. Furthermore, the obtained photovoltages were much higher than that predicted by the Schottky-Mott rule (0.61 V)^38^ owing to the formation of an interface dipole at the n-n BiVO_4_/Bi_4_V_2_O_11_ heterojunction, suggesting that the photovoltage generation by a simple n-n heterojunction is not the primary process governing the photovoltage.

### Mott-Schottky data

To gain more insights into the factors that improved the photovoltage, we investigated the electronic characteristics of the BiVO_4_/Bi_4_V_2_O_11_ heterojunction using electrochemical impedance spectroscopic, and the Mott-Schottky equation was used to evaluate the type of conductivity of the heterojunctions. Figure 5 shows the capacitance values of the space charge region obtained at various frequencies (1, 10 and 100 Hz). According to the Mott-Schottky equation, a linear relationship of 1/C^2^ *versus* applied potential achieved in Fig. 5 displayed straight lines with positive slopes for all the studied frequencies, which corresponds to depletion regions typical of n-type semiconductors. In this case, the photogenerated holes can be spontaneously driven to the BiVO_4_/Bi_4_V_2_O_11_ surface by the band bending in the space-charge layer, that is, where the electrode can act as a photoanode. Remarkably, in addition to the positive slopes, a region with a negative slope at sufficiently anodic voltages of the flatband potential was also observed in the W0 and W1 heterojunctions. It corresponds to a positively charged inversion layer, at which holes are the majority at the BiVO_4_/Bi_4_V_2_O_11_ interface. The inversion layer phenomenon was not detected in the single BiVO_4_ and Bi_4_V_2_O_11_, which suggests that this process may be occurring at the heterojunction interface. It reveals that in the inverted layer, the BiVO_4_/Bi_4_V_2_O_11_ heterojunctions can work as a photocathode. Therefore, the behavior of the films as photocathodes was also analyzed. Indeed, it was verified through the current-potential curves (Fig. S10) that both non-doped and W-doped BiVO_4_/Bi_4_V_2_O_11_ heterojunctions exhibited photoresponses that are typical of p-type semiconductors at cathodic potentials. The photocurrent values at 0 V_RHE_ for the W0 and W1 films were −0.03 and −0.14 mA cm^−2^, respectively. On the other hand, the pristine Bi_4_V_2_O_11_ and BiVO_4_ exhibited smaller photocurrent values of −0.004 and −0.010 mA cm^−2^ respectively, indicating that the p-type behavior of the photoelectrodes is typical of the heterojunction.

## Discussion

**Figure 1:**
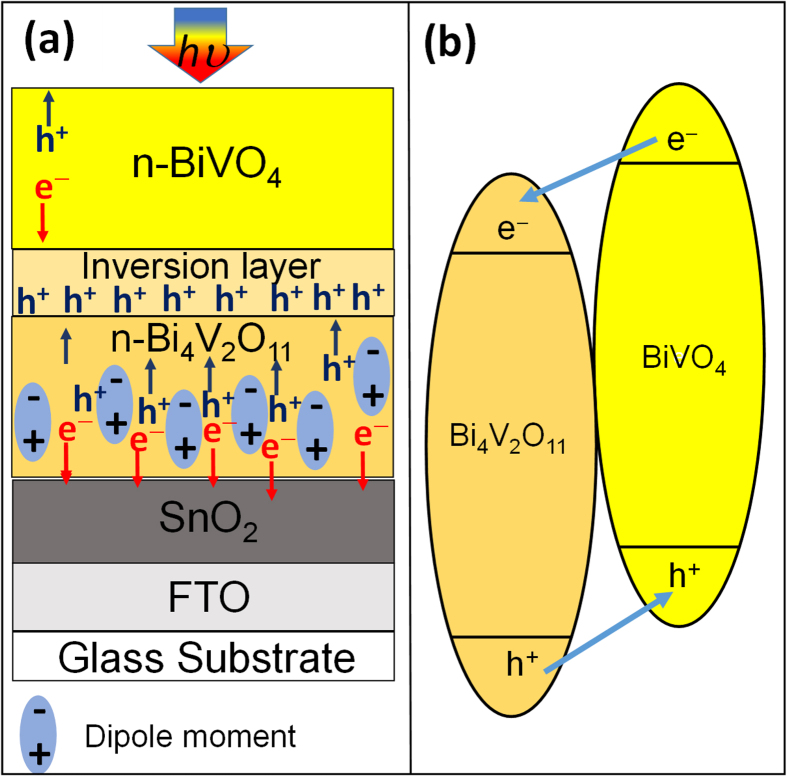
(**a**) The internal electrical field generated by the ferroelectric Bi_4_V_2_O_11_. Schematic drawing of n-BiVO_4_/ferroelectric n-Bi_4_V_2_O_11_ heterojunction with a polarization-induced inversion layer, and **(b**) Band alignment. Schematic illustration of BiVO_4_/Bi_4_V_2_O_11_ band alignment.

**Figure 2:**
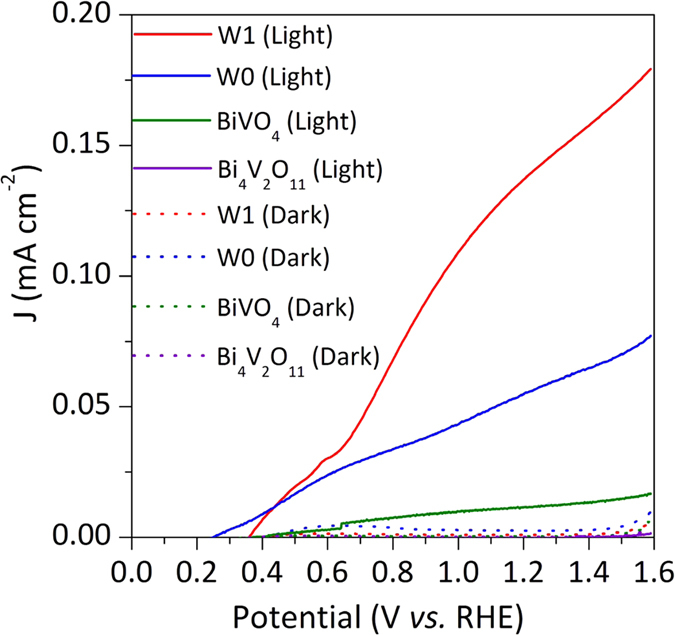
Photoelectrochemical characteristic of the photoelectrodes. Current-potential curves for the W0 and W1 films. Measurement conditions: active area of 1.0 cm^2^, 0.5 M Na_2_SO_4_ electrolyte (pH = 6.6), Light Source: White Light (λ > 450 nm, 5 mW cm^−2^), scan rate of 20 mV s^−1^ from low to high potential, front illumination for both photoanodes.

**Figure 3:**
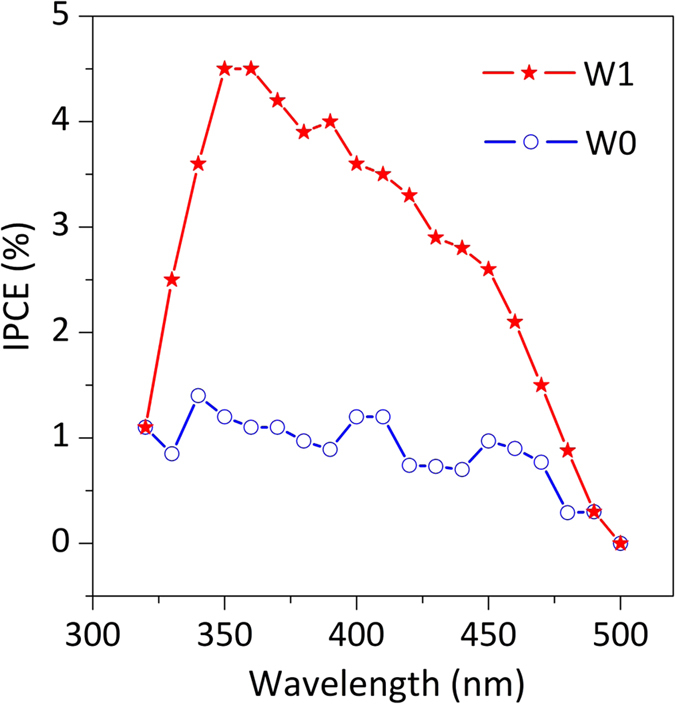
The incident photon-to-current efficiency. IPCE spectra of the W0 and W1 heterojunctions at 0 V vs. Pt in 0.5 M Na_2_SO_4_ electrolyte.

**Figure 4:**
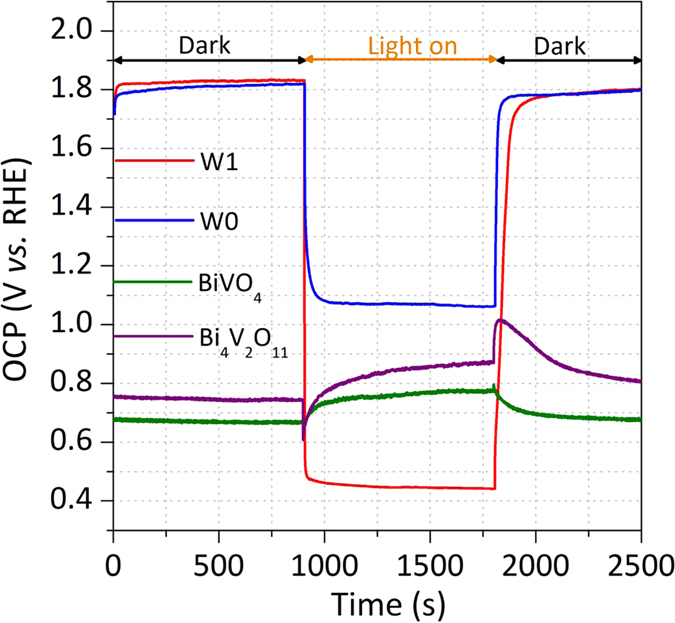
Photovoltage generated by the photoelectrodes. Open circuit potential measurements of BiVO_4_, Bi_4_V_2_O_11_, W0, and W1 photoelectrodes. Measurement conditions: active area of 1.1 cm^2^, 0.5 M Na_2_SO_4_ electrolyte (pH = 6.6), light source: white light (λ > 450 nm, 5 mW cm^−2^).

**Figure 5:**
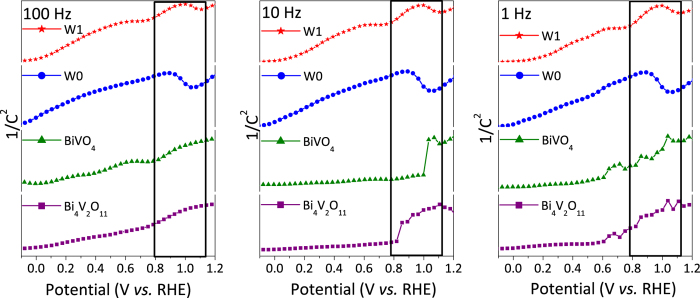
Inversion layer detected by Mott-Schottky plots. Variation of capacitance (C) with the applied potential in 0.5 M Na_2_SO_4_ (pH = 6.6) presented in the Mott-Schottky relationship for the films BiVO_4_, Bi_4_V_2_O_11_, W0, and W1 at different frequencies.

Given the above results, we suggest that the high photovoltages generated by the BiVO_4_/Bi_4_V_2_O_11_ heterojunctions may be assigned to the strong inversion layer at the BiVO_4_/Bi_4_V_2_O_11_ interface. The self-polarization due to the existence of (VO_3.5 0.5_)^2−^ layers in Bi_4_V_2_O_11_ creates a sufficiently high internal polar electric field to produce a hole inversion layer at the BiVO_4_/Bi_4_V_2_O_11_ interface, as verified from the Mott-Schottky data. The internal surface dipole on the Bi_4_V_2_O_11_ increases the local vacuum level at the heterojunction interface, thus changing the energy needed to extract an electron from the semiconductor into the vacuum and, therefore, can be thought of as altering the ionization potential, electron affinity, and the work function of the semiconductor. When the Bi_4_V_2_O_11_ containing dipole moments is brought into intimate contact with the BiVO_4_, it is the difference between the effective work functions of the two that is the driving force for achieving electronic equilibrium via charge transfer between them and the creation of a space charge region within the heterojunction. Thus, charges of the same polarity will be repelled from the heterojunction interface whereas those of the opposite polarity will be attracted to it (Fig. 1). Hence, if the ferroelectric Bi_4_V_2_O_11_ is in contact with the n-type BiVO_4_, majority electrons will be depleted from the BiVO_4_/Bi_4_V_2_O_11_ interface, while minority holes will be attracted to it, thus inverting its conductivity to p^+^ (p^+^ denotes highly doped semiconductors compared with p used for low/moderately doped one). As a consequence, a virtual p-n junction similar to the p-n junction occurring in a metal-insulating-semiconductor (MIS)^39^, can be observed. This is in agreement with the photoanodic and photocathodic behavior exhibited by the BiVO_4_/Bi_4_V_2_O_11_ heterojunction and Mott-Schottky data, which revealed typical characteristics of n- and p-type semiconductors. Because of that, photogenerated charges can be more efficiently separated by the virtual p-n heterojunction, thus providing high photovoltages. It is worth to note that the net photovoltage strongly depends on the polarization in Bi_4_V_2_O_11_, which can be efficiently tuned by creating oxygen vacancies and strain through W-doping into its structure. Additionally, the inversion layer has been shown to decrease the impact of surface trap states, thus reducing the surface recombination and consequently increasing the observed photocurrent^40^.

In summary, this work addresses a central topic on water splitting into PEC cells related to the low photovoltages generated by semiconductors. We have shown that the self-polarization of Bi_4_V_2_O_11_ perovskite creates an internal electrical field, which induces the formation of a hole inversion layer at the BiVO_4_/Bi_4_V_2_O_11_ interface. Because of that, a virtual p-n junction was formed at the BiVO_4_/Bi_4_V_2_O_11_ interface and consequently a high photovoltage was produced. The self-polarization effect was tuned by doping the ferroelectric material with W and, therefore, an increase of 83% in the generated photovoltage was developed as compared to the undoped heterojunction. Because the high photovoltage can promote an enhanced charge separation at the photoelectrodes, an increase of 180% in the photocurrent compared to the undoped heterojunction was achieved. We hope that the approach described here may enable the design of practical photoelectrodes based on earth-abundant semiconductors with high photovoltage and enhanced charge separation for water splitting.

## Methods

### Preparation of the precursor solution

All chemicals were used as received without further purification. Ten millimoles of ammonium metavanadate (NH_4_VO_3_, 99%) were dissolved in 50 mL of 174.2 mmol NH_4_OH (24.5%) to get the solution “A”. In another recipient, 10 mmol of Bi(NO_3_)_3_·5H_2_O was dissolved in 50 mL of 174.2 mmol CH_3_COOH (99.7%), producing the solution “B”. Both the solutions were stirred separately under heating at 80 °C to form stable and homogeneous solutions. Then, the solution A and B were mixed under stirring at 80 °C for 90 min. Next, 40 mmol citric acid was added to the mixture, and the resultant solution was kept standing for 24 h. The solution was then diluted with 100 mL deionized water and heated at 80 °C until the solution became light blue. This homogeneous solution was used as a precursor for preparing the photoelectrodes. Tungsten doped sample was achieved by a similar way as described above, except by the addition of ammonium metatungstate solution to the solution “A”.

### Preparation of the photoelectrodes

Transparent conductive FTO-coated glass (10 mm × 30 mm × 2 mm, 16 Ω cm^−2^) were used as substrates for the deposition of the films. Before the films deposition onto FTO, the substrate surface was cleaned in an ultrasonic bath using acetone and ethanol each for 15 min. After the cleaning procedure, the substrates were dried in a furnace at 120 °C for 1 h. Then, a SnO_2_ passivating layer was deposited onto FTO. Briefly, 0.4513 g SnCl_2_·2H_2_O was dissolved in 50 mL ethanol (99.8%) and 2 drops of CH_3_COOH (99.7%). This solution was sprayed down using a commercial airbrush (0.3 mm nozzle) directly onto the FTO glass onto FTO at 300 °C at a distance of 20 cm, with 2 cycles of spray deposition for 5 s each cycle. Films were subsequently annealed in air in a muffle furnace at 450 °C for 2 h, to produce a SnO_2_ layer. Finally, the precursor solutions were sprayed down onto the FTO glass at a temperature of 300 °C at a distance of 20 cm. 1 cycle of spray with 5 s time deposition was used. The films were subsequently annealed in air in a muffle furnace at 500 °C for 5 h to produce the undoped BiVO_4_/Bi_4_V_2_O_11_ (sample W0) and W-doped BiVO_4_/Bi_4_V_2_O_11_ (sample W1) photoelectrodes.

### Characterization

The morphology of films was investigated by scanning electron microscopy (SEM) using a tabletop SEM (Hitachi TM − 300). EDS mapping was obtained in a SwiftED3000 (Oxford Instruments) at 15 kV accelerating voltage. The crystalline phases of the films were determined using an X-ray diffractometer (XRD 6000, Shimadzu). The data were collected from 10 to 80^o^ 2θ at a step width of 0.2^o^, 10 s per step, at 40 kV, 200 mA, and CuKα radiation (λ = 1.540560 Å). Silicon was used as an external standard. The Rietveld structural refinement was performed with FullProf_Suite 2015 software. The diffuse reflectance spectra were collected with a UV-Vis spectrophotometer (Shimadzu UV 2700). Teflon was used as reference material (100% transmission). The direct bandgap energies were calculated by the following Tauc equation (1):


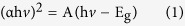


where A = constant, hν = light energy, E_g_ = optical bandgap energy, α = measured absorption coefficient.

### Photoelectrochemical measurements

The photoelectrochemical measurements were carried out with a potentiostat (AUTOLAB Potentiostat-Galvanostat PGSTAT 128 N) using a standard three-electrode cell with an Ag/AgCl (3.0 M KCl) reference electrode, a platinum wire as a counter electrode, a working electrode with irradiation area of 1.1 cm^2^, and a scan rate of 20 mV s^−1^. A 0.5 M Na_2_SO_4_ aqueous solution (pH = 6.6) was used as electrolyte. The prepared films were connected to a copper tape to measure the photoactivity. The current-potential curves were recorded in the dark and under a white light LED (light intensity of 5 mW cm^−2^, λ > 450 nm). For converting the obtained potential *vs.* Ag/AgCl to RHE, the equation (2) was used:


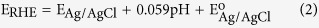


The E^o^_Ag/AgCl_ (3 M KCl) = 0.197 at 25 °C.

### Electrochemical impedance spectroscopy

The electrochemical impedance spectroscopy was performed using an AUTOLAB Potentiostat-Galvanostat PGSTAT 128 N equipped with the FRA32M module. The Nyquist plots were measured at 0.7 V *vs.* Ag/AgCl with an AC amplitude of 20 mV, frequency of 100 kHz−100 mHz under a white light LED ((light intensity of 5 mW cm^−2^, λ > 450 nm). The measured spectra were fitted using the NOVA 1.11 software. The 0.5 M Na_2_SO_4_ solution was utilized for the all electrochemical measurements.

Mott-Schottky data acquisition is a usual way for the electrochemical characterization of semiconductor materials. The spectra were collected using an AUTOLAB Potentiostat-Galvanostat PGSTAT 128 N equipped with the FRA32M module, and a cell configuration with three electrodes: a reference electrode of Ag/AgCl (3, 0 M KCl), a platinum as counter electrode wire and a working electrode (W0 or W1) with 1.0 cm^2^ irradiation area under white light LED (light intensity of 5 mW cm^−2^, λ > 450 nm), applying potential −0.6 V to +0.65 V *vs.* Ag/AgCl in the frequency range of 1 to 100 Hz. The measured spectra were fitted using the NOVA 1.11 software. The 0.5 M Na_2_SO_4_ solution was utilized for the all electrochemical measurements.

### IPCE measurements

Incident photon-to-current efficiency (IPCE) spectra were determined from photocurrent measurements, and the equation (3) was used:


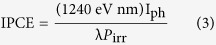


where, I_ph_ is the photocurrent density in mA cm^−2^, l is the irradiation wavelength of the incident light in nm, and P_irr_ is the incident photon flux in mW cm^−2^. In these experiments, the excitation was obtained by a 300 W xenon lamp in a Newport/Oriel housing, which was passed through a Newport/Oriel Cornerstone 260 monochromator to produce monochromatic light with a bandwidth of 10 nm. Photon flux was measured with a Newport 1919-R power meter equipped with an 818-UV/DB sensor before and after photocurrent measurements to ensure stability and reproducibility. Photo-action spectra were acquired at 10 nm intervals between 320 and 500 nm for the bared and W-doped BiVO_4_/Bi_4_V_2_O_11_ films immersed in 0.5 M Na_2_SO_4_ solution and using a platinized FTO as the counter electrode. The photoanode and the counter-electrode were mounted in a sandwich-like setup, using an inert polymer (50 μm Dupont Surlyn) as a spacer. The space between the electrodes was fulfilled by the electrolyte using a vacuum.

## Additional Information

**How to cite this article**: dos Santos, W. S. *et al*. A hole inversion layer at the BiVO_4_/Bi_4_V_2_O_11_ interface produces a high tunable photovoltage for water splitting. *Sci. Rep.* **6**, 31406; doi: 10.1038/srep31406 (2016).

## Supplementary Material

Supplementary Information
