# Supplementary material for: A hole inversion layer at the BiVO4/Bi4V2O11 interface produces a high tunable photovoltage for water splitting
Source: Sci Rep. 2016 Aug 9;6:31406. doi: 10.1038/srep31406 (PMC4977555; doi:10.1038/srep31406)
Supplement: Supplementary Information [file srep31406-s1.pdf]

Electronic Supplementary Information

A hole inversion layer at the $BiVO_4/Bi_4V_2O_{11}$ interface produces a high tunable photovoltage for water splitting

Wayler S. dos Santos,[1,2] Mariandry Rodriguez,[1] André S. Afonso,[1] João P. Mesquita,[3] Lucas L. Nascimento,[4] Antonio O. T. Patrocínio,[4] Adilson C. Silva,[5] Luiz C. A. Oliveira,[6] José D. Fabris[2] & Márcio C. Pereira[1,*]

[1]*Institute of Science, Engineering and Technology (ICET), Federal University of the Jequitinhonha and Mucuri Valleys, Campus Mucuri, Teófilo Otoni, Minas Gerais, 39803-371, Brazil.*

[2]*Graduate Program in Biofuels, Federal University of the Jequitinhonha and Mucuri Valleys, Campus JK, 39100-000, Diamantina, Minas Gerais, Brazil.*

[3]*Department of Chemistry, Federal University of the Jequitinhonha and Mucuri Valleys, Campus JK, 39100-000, Diamantina, Minas Gerais, Brazil.*

[4]*Institute of Chemistry, Federal University of Uberlândia, 38400-902, Uberlândia, Minas Gerais, Brazil.*

[5]*Institute of Exact and Biological Sciences, Federal University of Ouro Preto, 35400-000, Ouro Preto, Minas Gerais, Brazil.*

[6]*Department of Chemistry, Federal University of Minas Gerais, 31270-901 Belo Horizonte, Minas Gerais, Brazil.*

***Corresponding author:** Tel./Fax: +55 33 35292700; Email address: mcpqui@gmail.com (M.C. Pereira)

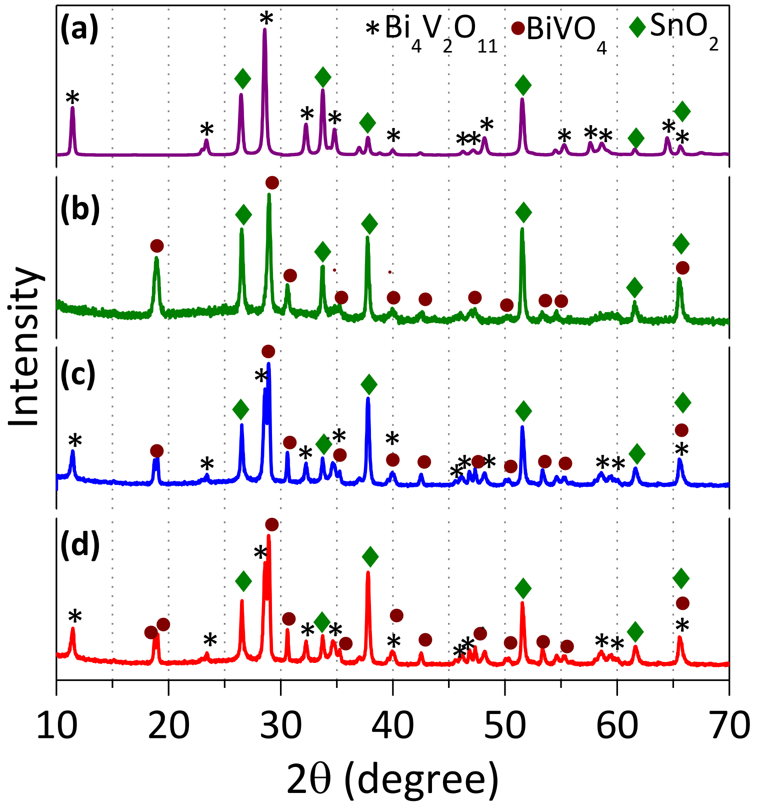

Figure S1. XRD patterns of the samples (a) $Bi_4V_2O_{11}$, (b) $BiVO_4$, (c) W0, and (d) W1.

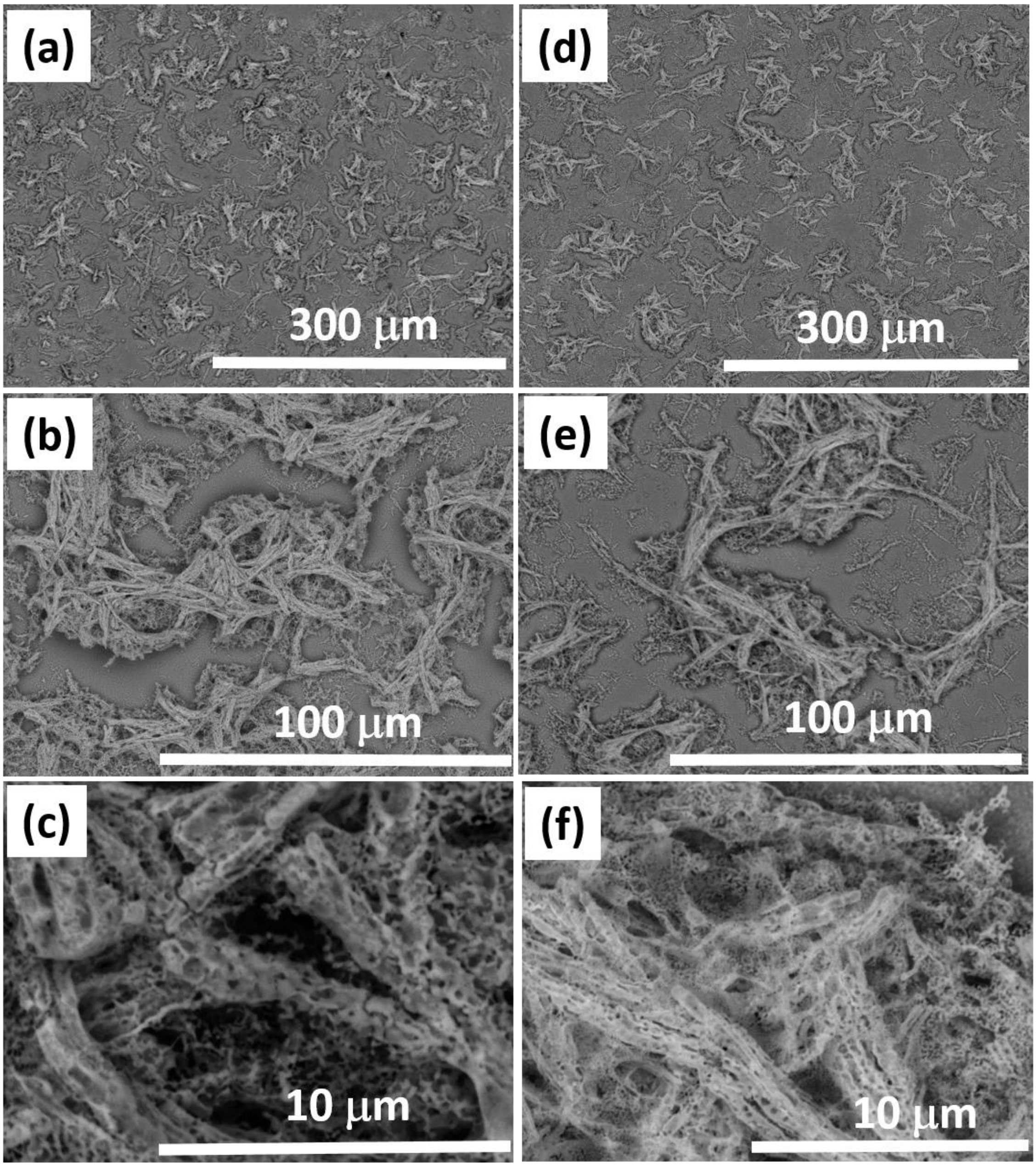

Figure S2. SEM images obtained for the samples (a-c) W0 and (d-f) W1.

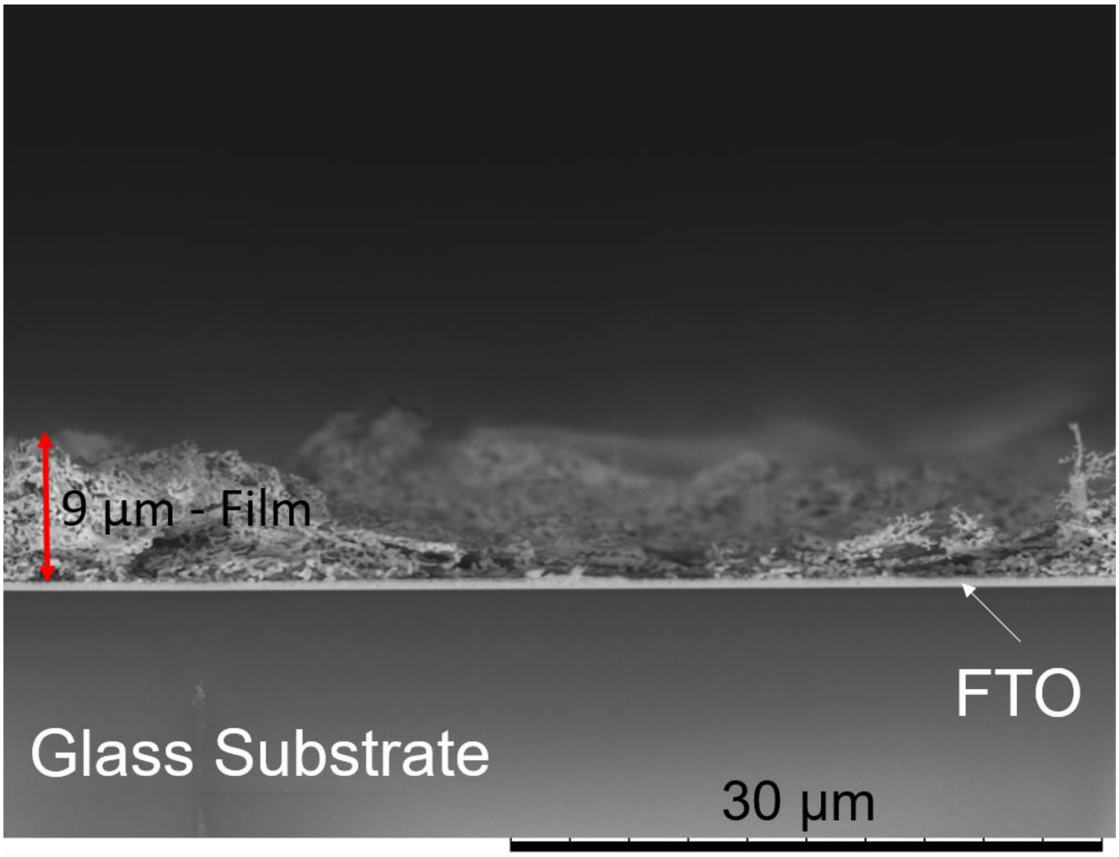

Figure S3. Cross-sectional SEM image of the sample W1 showing the film thickness.

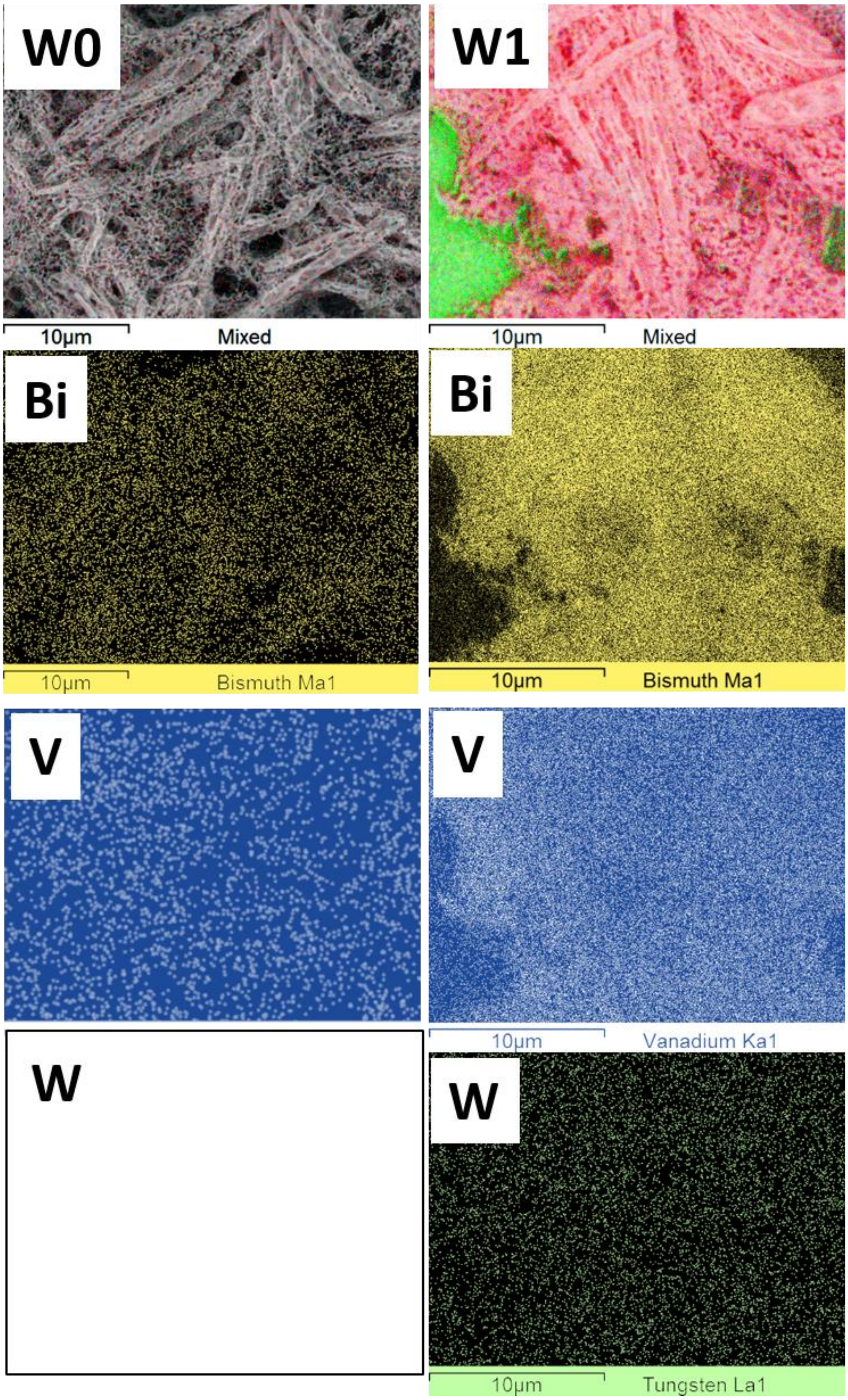

Figure S4. EDS mapping images of the films W0 and W1.

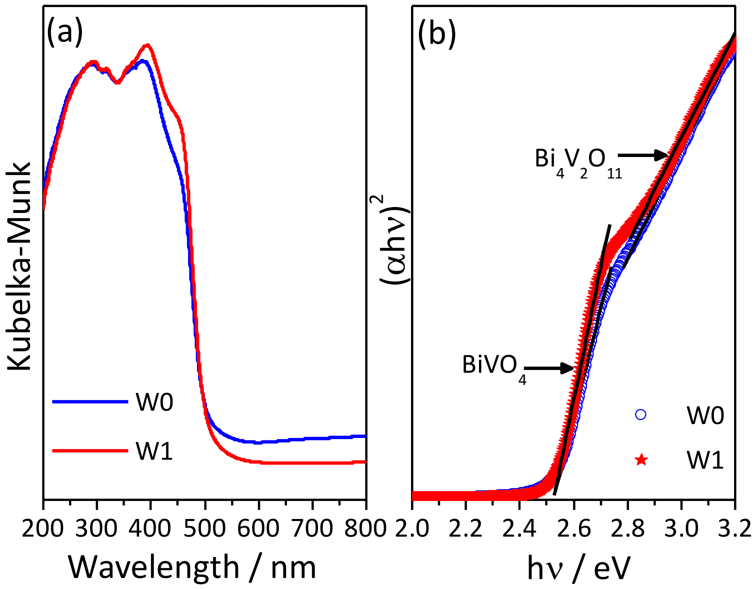

Figure S5. (a) UV-Vis diffuse reflectance spectra and (b) Tauc's plot of the W0 and W1 films.

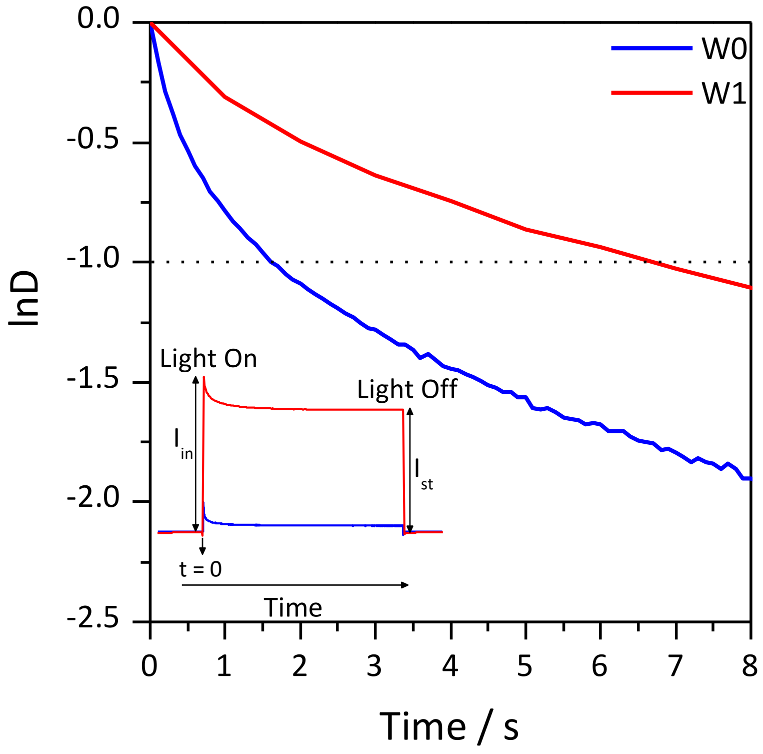

Figure S6. Normalized plots of the photocurrent-time dependence for W0 and W1 films. It was normalized by defining the parameter as $D = (I_{(t)} - I_{(st)})/(I_{(in)} - I_{(st)})$, where $I_{(t)}$ is the photocurrent at a time t, $I_{(in)}$ is the initial photocurrent at t = 0, and $I_{(st)}$ is the steady-state photocurrent. The transient time constant, τ, is defined as the time at which lnD = −1.[2] The inset represents a typical photocurrent transient response at a constant potential of 1.23 V_{RHE}.

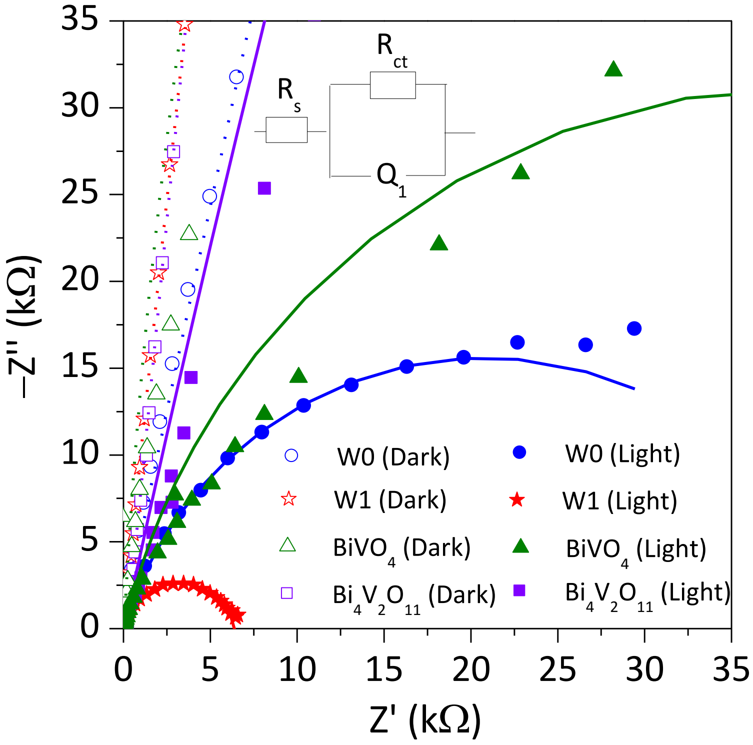

Figure S7. Electrochemical impedance spectra of the photoelectrodes in a 0.5 M Na_2SO_4 electrolyte. In the equivalent circuit (inset), R_s represents the circuit series-resistance, Q1 is the capacitance phase element of the semiconductor-electrolyte interface, and R_{ct} is the charge transfer resistance across the interface. Light source: White LED (5 mW cm^{-2}, λ > 450 nm); Frequency: 100 mHz-10 kHz.

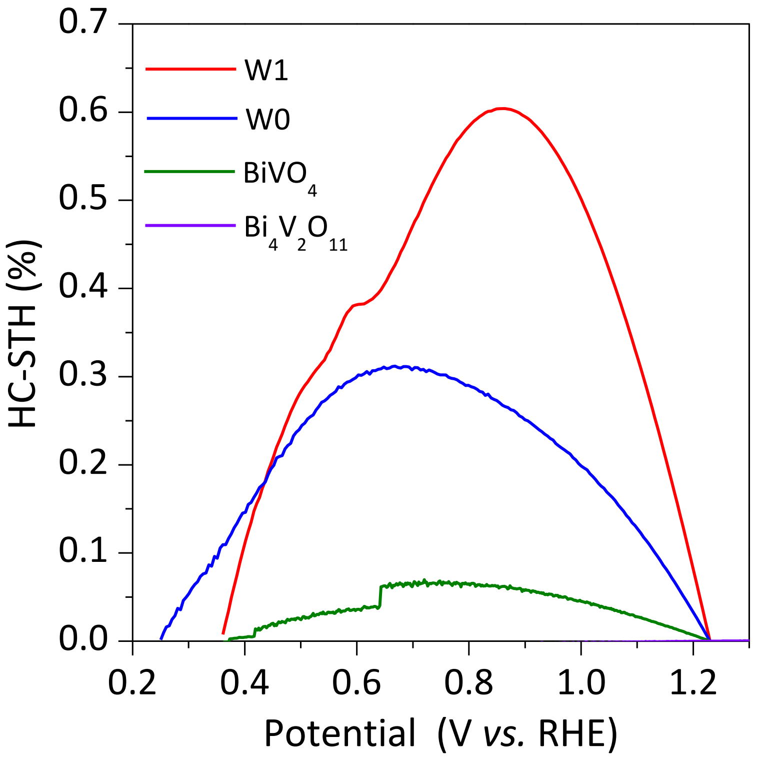

Figure S8. Half-cell solar-to-hydrogen efficiency (HC-STH) of the W0 and W1 photoanodes.

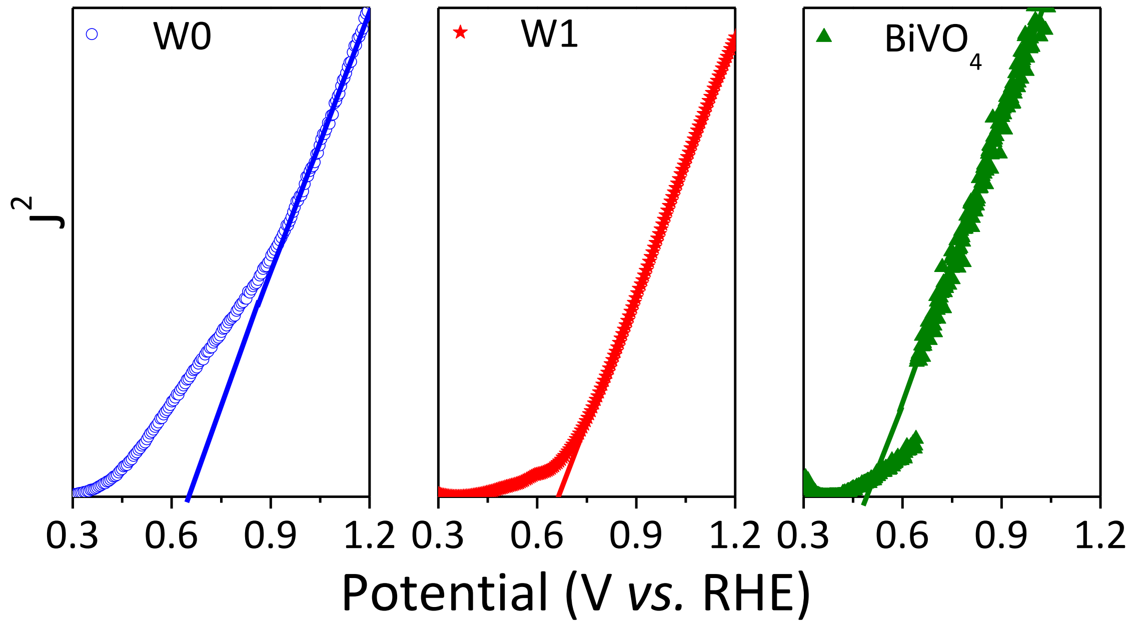

Figure S9. Onset potential determination from J^2 x applied potential dependence for the W0, W1, and $BiVO_4$ photoanodes. Light source: White LED (5 mW cm^{-2}, $\lambda > 450$ nm); electrolyte: 0.5 M Na_2SO_4 (pH = 6.6).

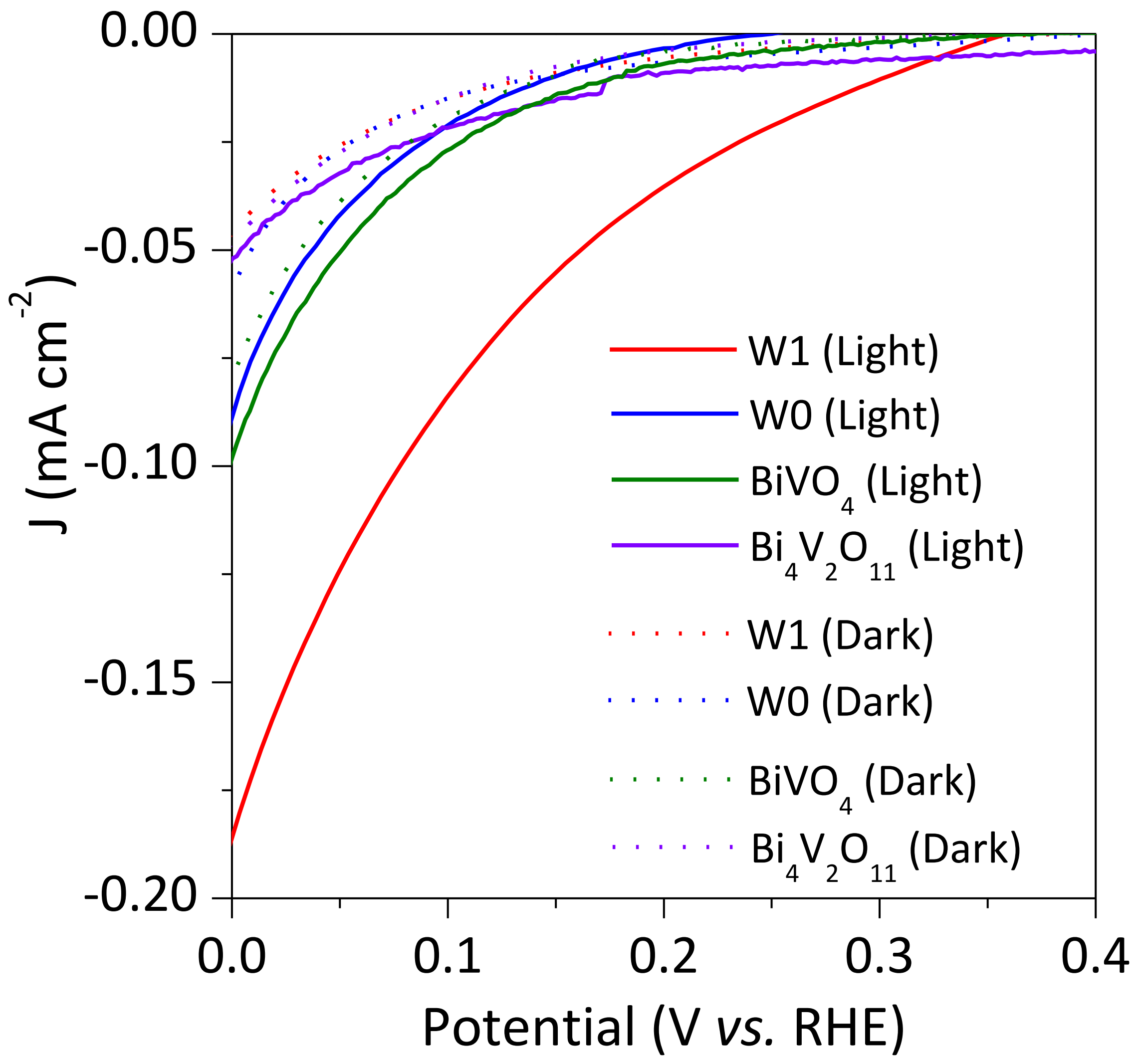

Figure S10. Current-potential curves for the W0 and W1 films. Measurement conditions: active area of 1.1 cm2, 0.5 M Na_2SO_4 electrolyte (pH = 6.6), light source: White light (λ > 450 nm, 5 mW cm^{-2}), scan rate of 20 mV s^{-1} from low to high potential, front illumination for both photoelectrodes.

Supplementary References

1. S. U. M. Khan and J. Akikusa. *J. Phys. Chem. B*, 1999, **103**, 7184-7189.
2. D. Tafalla, P. Salvador and R. M. Benito. *J. Electrochem. Soc.*, 1990, **137**, 1810-1815.
3. M. K. Brennaman, A. O. T. Patrocinio, W. Song, J. W. Jurss, J. J. Concepcion, P. G. Hoertz, M. C. Traub, N. Y. Murakami Iha, and T. J. Meyer. *ChemSusChem* 2011, **4**, 216-227.
